# Identification of an m6A-Related Signature as Biomarker for Hepatocellular Carcinoma Prognosis and Correlates with Sorafenib and Anti-PD-1 Immunotherapy Treatment Response

**DOI:** 10.1155/2021/5576683

**Published:** 2021-06-10

**Authors:** Hongye Jiang, Gang Ning, Yensheng Wang, Weibiao Lv

**Affiliations:** ^1^Department of Clinical Laboratory, Shunde Hospital, Southern Medical University (The First People's Hospital of Shunde), Foshan, Guangdong Province, China; ^2^Department of Gastroenterology and Hepatology, Guangzhou Digestive Diseases Center, Guangzhou First People's Hospital, South China University of Technology, Guangzhou, Guangdong Province, China; ^3^Department of Medicine, Chang-Gung Memorial Hospital, Linkou, Taiwan, China

## Abstract

**Background:**

N6-methyladenosine (m6A) modification plays an essential role in diverse key biological processes and may take part in the development and progression of hepatocellular carcinoma (HCC). Here, we systematically analyzed the expression profiles and prognostic values of 13 widely reported m6A modification-related genes in HCC.

**Methods:**

The mRNA expression of 13 m6A modification-related genes and clinical parameters of HCC patients were downloaded from TCGA, ICGC, GSE109211, and GSE78220. Univariate and LASSO analyses were used to develop risk signature. Time-dependent ROC was performed to assess the predictive accuracy and sensitivity of risk signature.

**Results:**

FTO, YTHDC1, YTHDC2, ALKBH5, KIAA1429, HNRNPC, METTL3, RBM15, YTHDF2, YTHDF1, and WTAP were significantly overexpressed in HCC patients. YTHDF1, HNRNPC, RBM15, METTL3, and YTHDF2 were independent prognostic factors for OS and DFS in HCC patients. Next, a risk signature was also developed and validated with five m6A modification-related genes in TCGA and ICGC HCC cohort. It could effectively stratify HCC patients into high-risk patients with shorter OS and DFS and low-risk patients with longer OS and DFS and showed good predictive efficiency in predicting OS and DFS. Moreover, significantly higher proportions of macrophages M0 cells, neutrophils, and Tregs were found to be enriched in HCC patients with high risk scores, while significantly higher proportions of memory CD4 T cells, gamma delta T cells, and naive B cells were found to be enriched in HCC patients with low scores. Finally, significantly lower risk scores were found at sorafenib treatment responders and anti-PD-1 immunotherapy responders compared to that in nonresponders, and anti-PD-1 immunotherapy-treated patients with lower risk scores had better OS than patients with higher risk scores.

**Conclusion:**

A risk signature developed with the expression of 5 m6A-related genes could improve the prediction of prognosis of HCC and correlated with sorafenib treatment and anti-PD-1 immunotherapy response.

## 1. Introduction

Hepatocellular carcinoma (HCC) is a common type of cancer and represents the leading cause of cancer-related death worldwide. HCC is still a serious burden to public health [1]. There were about 841,000 patients developed HCC, and 782,000 patients died from HCC alone in 2018 because of late diagnosis and limited treatment options [1, 2]. Moreover, the incidence of HCC is increasing rapidly with 50% recurrence rate after surgical treatment [3, 4]. It is well recognized that development and progression of HCC is the result of multistep process, where interactions between genetics and epigenetics have played important roles [5–8]. Understanding the pathogenesis of HCC is the key to discover new diagnostic biomarkers and therapeutic targets.

RNA modification, discovered in the 1970s, has recently been recognized as a third layer of epigenetics that could modify a plethora of native cellular RNAs [9–11]. N6-methyladenosine (m6A) modification is the most abundant form of internal mRNA methylation among the kinds of RNA modifications in eukaryotes [12]. m6A modifications in mammalian cells are dynamic and reversible and are commonly regulated by binding proteins (“readers”), methyltransferases (“writers”), and demethylases (“erasers”) [13]. Among m6A modification-related genes, 13 genes, including *ZC3H13*, *WTAP*, *KIAA1429*, *METTL3*, *METTL14*, *RBM15*, *YTHDC1*, *YTHDC2*, *YTHDF1*, *YTHDF2*, *HNRNPC*, *ALKBH5*, and *FTO*, are the most prominent [14–16]. These m6A modification-related genes are primarily involved in modulation of alternative mRNA splicing, precession of pre-miRNA, stability of mRNA, and enhancement of translation efficiency of mRNA [13]. Not only do these 13 m6A modification-related genes play essential roles in many important biological processes, such as development of embryonic and neural cells, differentiation of stem cell, and stress responses [17–19], they also take part in the development, progression, and radio resistance of various kinds of cancers [20–23]. For example, overexpression of *YTHDF1* is found to be related with poorer survival of HCC patients, and *KIAA1429* and *METTL3* are found to regulate migration and invasion of HCC, indicating an important role of m6A modification-related genes playing in HCC [24–26].

Recently, Zhou et al. explored the expression pattern and prognostic values of m6A modification-related genes of HCC patients, but they mainly focused on the role of *METTL3* and *YTHDF1* [27]. In the present study, we comprehensively analyzed the expression pattern and prognosis of the thirteen widely reported m6A modification-related genes in TCGA HCC cohort. Besides, we also developed and validated a risk signature with the expression of 5 selected m6A modification-related genes and analyzed its prognostic value for HCC patients and its relation with tumor-infiltrating immune cells in TCGA and ICGC HCC cohort. Moreover, the prediction values of risk signature in sorafenib treatment and anti-PD-1 immunotherapy response were also evaluated.

## 2. Materials and Methods

### 2.1. Ethics Statement

All the data analyzed in the present study were received from TCGA, ICGC, and GEO dataset, and written consents were already obtained before our study.

### 2.2. Data Collection

mRNA expression of TCGA HCC cohorts, which included 374 HCC cases and 50 normal controls, was got from GDC Data Portal (https://cancergenome.nih.gov/). Meanwhile, corresponding clinical-pathological data, including gender, age, histologic grade, tumor T stage, N stage, M stage (M), TNM stage, overall survival (OS) time, and disease-free survival (DFS) time, were also downloaded. It was of note that 9 of 374 HCC patients were excluded because of absence of corresponding clinical-pathological data, and basic characteristics of 365 HCC patients were summarized in Table 1. In addition, a total of 232 HCC patients with available OS information and mRNA expression were got from the ICGC portal (https://dcc.icgc.org/projects/LIRI-JP). The mRNA expression of 67 sorafenib-treated HCC patients of GSE109211 was downloaded from the GEO database (https://www.ncbi.nlm.nih.gov/geo/), and there were 21 sorafenib treatment responders and 46 nonresponders in GSE109211. Moreover, the mRNA expression of 27 melanoma patients with anti-PD-1 checkpoint inhibition therapy of GSE78220 was also downloaded from the GEO database. Four patients achieved complete response, 10 patients achieved partial response, and 13 patients achieved no response.

### 2.3. Development and Validation of Risk Signature

First, univariate analysis was carried out to select the genes related with survival. Then LASSO algorithm was used for selecting the most prognostic-related genes [28]. A risk signature was developed based on the coefficients weighted by LASSO analysis. With this signature, we calculated a risk score for HCC patients and divided HCC patients into high-risk group and low-risk group based on the median risk score.

### 2.4. CIBERSORT

CIBERSORT (https://cibersort.stanford.edu) is an online tool designed for estimating the abundances of 22 kinds of tumor-infiltrating immune cells with transcriptomic data [29], and we used it to calculate the tumor-infiltrating immune cells of HCC patients basing on the mRNA expression profiles of TCGA HCC cohort and ICGC HCC cohort, respectively.

### 2.5. Data Analysis Flow Chart

**Figure 1:**
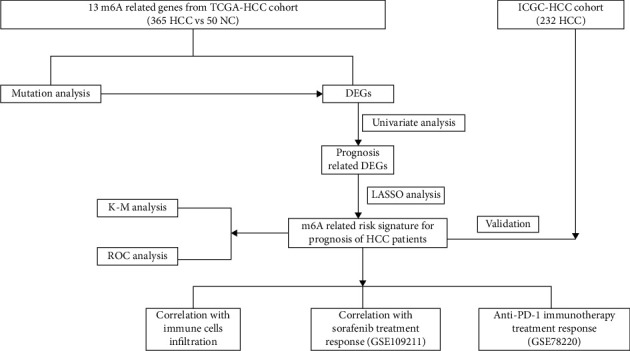
The workflow chart of the present study.

To make the study to be better understood, a workflow of the study was depicted and was shown at Figure 1.

### 2.6. Statistical Analysis

The R software (version 3.5.1) was used for statistical analysis. Wilcox test was performed to compare difference of m6A modification-related genes between HCC and healthy controls. Correlation of the 13 m6A modification-related genes with each other was compared by Spearman correlation analysis. One-way ANOVA was carried out to compare difference of m6A modification-related genes among different histologic grades and TNM stages. Chi-square analysis was carried out to analyze distribution of clinical-pathologic parameters between high-risk HCC patients and low-risk HCC patients. Univariate and multivariate Cox regression analyses were carried out to analyze the prognostic value of m6A modification-related genes and risk signature. Kaplan-Meier analysis with log-rank test was carried out to analyze difference of OS or DFS between patients of different clusters or with risk scores. Time-dependent ROC was carried out to analyze the predictive accuracy and sensitivity of risk signature. Additional statistical analyses were performed with STAMP [30]. *P* < 0.05 was considered as statistically significant.

## 3. Results

### 3.1. Expression of m6A Modification-Related Genes of HCC Patients and Their Associations with Clinical-Pathologic Parameters

**Figure 2:**
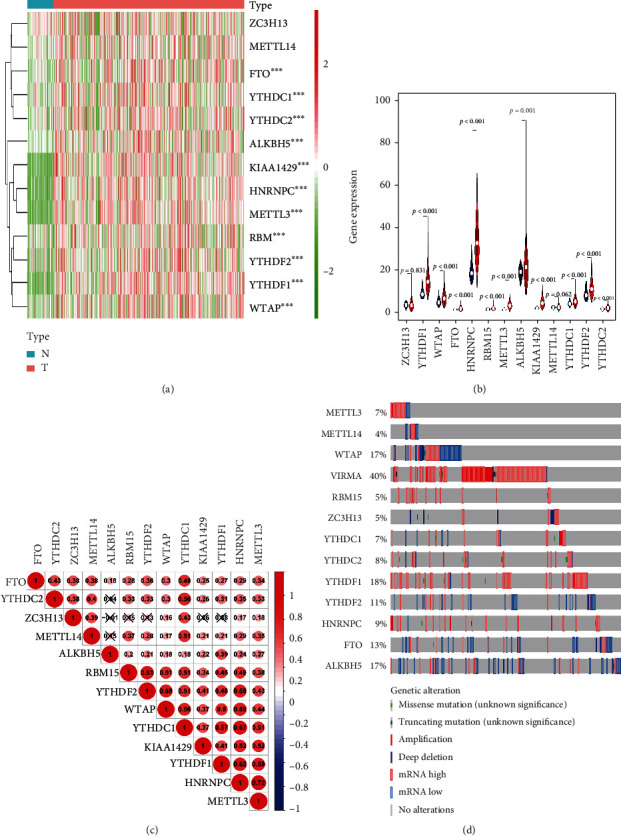
Expression of 13 m6A modification-related genes in HCC and their associations with clinical-pathologic parameters. (a) Heatmap of log2 transformed expression of 13 m6A modification-related genes between HCC patients and normal controls. (b) Violin plot of expression of 13 m6A modification-related genes between HCC patients and normal controls. (c) Correlation of the 13 m6A modification-related genes with each other. (d) Genetic changes of the 13 m6A modification-related genes. ^∗^*P* < 0.05, ^∗∗^*P* < 0.01, and ^∗∗∗^*P* < 0.001.

First, the mRNA expression of 13 m6A modification-related genes was downloaded from TCGA and compared between HCC patients and normal controls. As was shown at Figures 2(a) and 2(b), significantly higher expression of *FTO*, *YTHDC1*, *YTHDC2*, *ALKBH5*, *KIAA1429*, *HNRNPC*, *METTL3*, *RBM15*, *YTHDF2*, *YTHDF1*, and *WTAP* was found in the tissues of HCC patients compared to normal tissues (all *P* < 0.001). Interestingly, we also found that the expression of most of the 13 m6A modification-related genes seemed to be lower than those of other 32 kinds of tumors. Besides, most of the 13 m6A modification-related genes were positively correlated with each other (Figure 2(c)). Moreover, genetic changes, such as missense mutation, truncating mutation, amplification, deep deletion, diploid, and gain, were observed in about 80% of the HCC patients (Figure 2(d)). Specifically, each HCC patient might have one or more kinds of genetic changes. The genetic rates of *WTAP*, *KIAA1429*, *RBM15*, *METTL3*, *METTL14*, *ALKBH5*, *YTHDC1*, *YTHDC2*, *HNRNPC*, *YTHDF1*, *YTHDF2*, *FTO*, and *ZC3H13* were 7%, 4%, 17%, 40%, 5%, 5%, 7%, 8%, 18%, 11%, 9%, 13%, and 17%, respectively, suggesting that higher expression of m6A modification-related genes might be the result of genetic changes in related genes. Taken together, these results indicated that m6A modification-related genes played important roles in HCC.

### 3.2. Prognostic Value of m6A Modification-Related Genes in HCC Cases

Next, we further analyzed prognostic values of m6A modification-related genes. Univariate analysis showed that higher expression of *YTHDF1*, *WTAP*, *HNRNPC*, *RBM15*, *METTL3*, *KIAA1429*, *YTHDC1*, and *YTHDF2* and lower expression of *ZC3H13* were statistically related to poorer OS of HCC patients (all *P* < 0.05, supplementary figure 1A); multivariate analysis showed that the expression of *YTHDF1*, *WTAP*, *HNRNPC*, *RBM15*, *METTL3*, *KIAA1429*, and *YTHDF2* still remained significantly related with OS after adjusting for gender, age, histologic grade, T stage, N stage, M stage, and TNM stage (all *P* < 0.05, supplementary figure 1B-1J). Then, the prognostic values of m6A modification-related genes for recurrence of HCC patients were also analyzed. Univariate analysis indicated that overexpression of *YTHDF1*, *WTAP*, *HNRNPC*, *RBM15*, *METTL3*, *YTHDC1*, and *YTHDF2* was statistically related with shorter DFS (all *P* < 0.05, supplementary figure 2A); multivariate analysis showed that the expression of *YTHDF1*, *HNRNPC*, *RBM15*, *METTL3*, and *YTHDF2* was still statistically related with DFS after adjusting for gender, age, histologic grade, T stage, N stage, M stage, and TNM stage (all *P* < 0.05, supplementary figure 2B-2H). These results strongly confirmed the important roles played by m6A modification-related genes in HCC.

### 3.3. Development of Risk Signature with 5 m6A Modification-Related Genes and Its Association with Clinical-Pathologic Parameters

**Figure 3:**
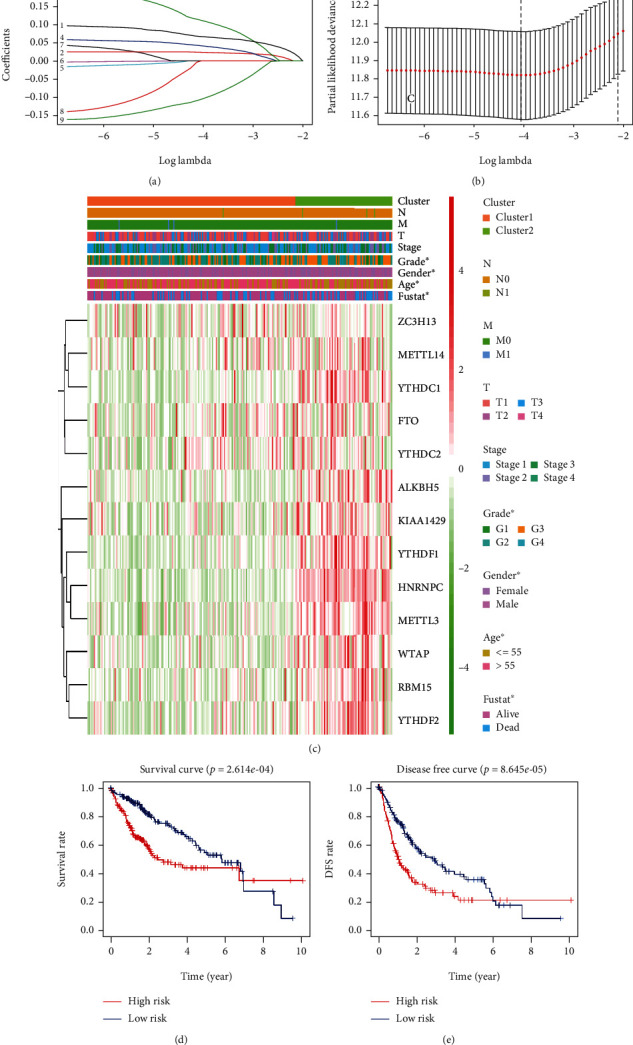
Construction of risk signature with 5 m6A modification-related genes and its association with clinical parameters. (a, b) 5 m6A modification-related genes identified by LASSO analysis. (c) Heatmap of the association of risk score with clinical-pathologic parameters. (d) Kaplan-Meier analysis of OS of patients of high-risk subgroup and low-risk subgroup. (e) Kaplan-Meier analysis of DFS of patients of high-risk subgroup and low-risk subgroup. T: tumor stage; N: lymph node stage; M: metastasis stage; stage: TNM stage; ^∗^*P* < 0.05, ^∗∗^*P* < 0.01, and ^∗∗∗^*P* < 0.001.

To better explore the prognostic value of m6A modification-related genes, a risk signature was developed. Based on the results of univariate analysis (Figure 3(a)), *ZC3H13*, *YTHDF1*, *WTAP*, *HNRNPC*, *RBM15*, *METTL3*, *KIAA1429*, *YTHDC1*, and *YTHDF2* were associated with OS and were considered as prognostic-related genes. Then, LASSO analysis was used to further screen the prognostic-related genes. In the end, 5 genes, including *YTHDF2*, *YTHDF1*, *METTL3*, *KIAA1429*, and *ZC3H13*, were used to develop the risk signature (Figures 3(a) and 3(b)). The risk score was then constructed based on the coefficients weighted by LASSO analysis and calculated as follows: risk score = (0.07∗*YTHDF*2) + (0.02∗*YTHDF*1) + (0.11∗*METTL*3) + (0.04∗*KIAA*1429) − (0.1∗*ZC*3*H*13). We calculated the risk score for every HCC case and assigned them into high-risk group and low-risk group on the basis of the median risk score. The expression of *YTHDF2*, *YTHDF1*, *METTL3*, and *KIAA1429* tended to be higher in patients with high risk score; the expression of *ZC3H13* seemed to be higher in patients with low risk score (Figure 3(c)). Distribution of histologic grade, T stage, and TNM stage was significantly different between high-risk subgroup and low-risk subgroup (all *P* < 0.05, Figure 3(c)). High-risk subgroup contained more patients with advanced histologic grade, T stage, and TNM stage compared to patients of the low-risk subgroup. Lastly, patients in the high-risk subgroup had poorer OS (median OS time: 2.46 vs. 5.79 years, HR = 1.98, 95% CI: 1.39-2.83, and *P* < 0.001; Figure 3(d)) and shorter DFS (median DFS: 1.07 vs. 2.97 years, HR = 3.83, 95% CI: 2.56-5.90, and *P* < 0.001; Figure 3(e)) than those of patients of the low-risk subgroup, which were consistent with the previous results.

### 3.4. Prognostic Value of Risk Signature for OS and DFS of HCC Cases

**Figure 4:**
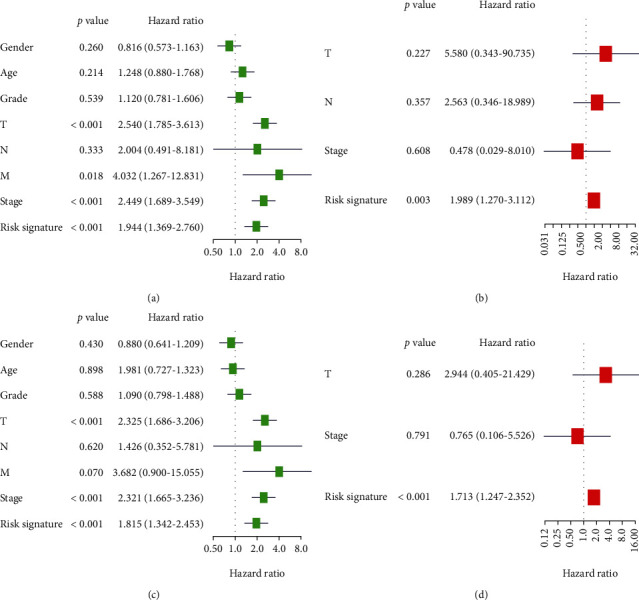
Prognostic value of risk signature for OS and DFS of HCC patients. (a) Univariate analysis of risk signature with OS of HCC patients. (b) Multivariate analysis of risk signature with OS of HCC patients. (c) Univariate analysis of risk signature with DFS of HCC patients. (d) Multivariate analysis of risk signature with DFS of HCC patients. Gender: male vs. female; age: >60 vs. ≤60; grade: G3+G4 vs. G1+G2; T: T1 vs. T0; N: N1 vs. N0; M: M1 vs. M0; TNM stage: stage III+IV vs. stage I+II.

The risk signature was found to be associated with clinical-pathologic parameters. We next performed univariate and multivariate analyses to analyze its prognostic value. Based on the univariate analysis, T stage, M stage, TNM stage, and risk signature were statistically related with OS of HCC patients (all *P* < 0.05, Figure 4(a)). The risk signature still remained statistically related with OS after adjusting for T stage, M stage, and TNM stage by multivariate analysis. In multivariate analysis, after adjusting for TNM stage, the risk signature was still significantly related with OS (*P* < 0.01, Figure 4(b)). Similarly, univariate analysis also showed that T stage, TNM stage, and risk signature were statistically related with DFS of HCC patients. In univariate analysis, T stage, TNM stage, and the risk signature were also significantly associated with DFS in HCC patients (all *P* < 0.001, Figure 4(c)). By incorporating these factors into multivariate analysis, the result suggested that only the risk signature was statistically related with DFS (*P* < 0.001, Figure 4(d)). To conclude, these results indicated that the risk signature was an independent prognostic factor for OS and DFS of HCC patients.

**Figure 5:**
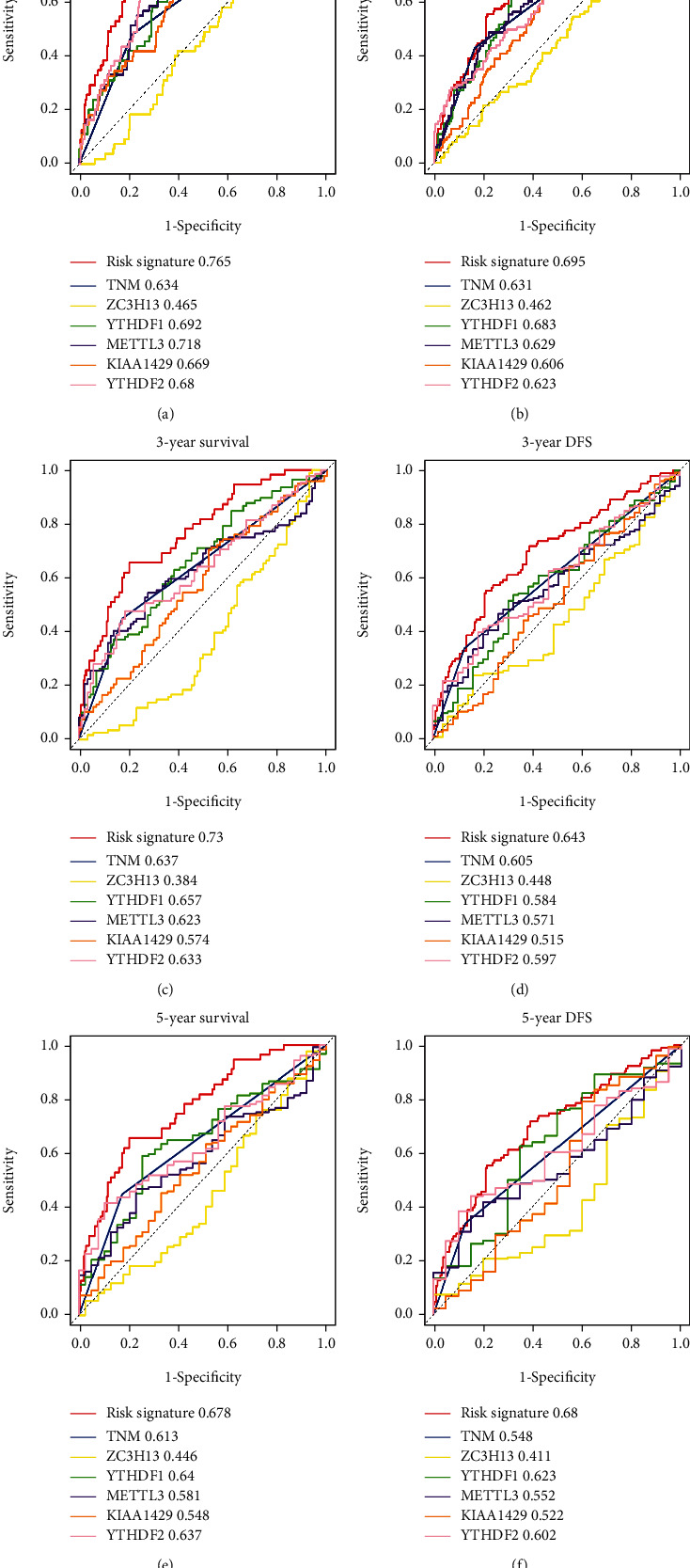
Predictive value of risk signature, TNM stage, *YTHDF2*, *YTHDF1*, *METTL3*, *KIAA1429*, and *ZC3H13*. Time-dependent ROC analysis was used to evaluate the predictive value in predicting (a) 1-year, (c) 3-year, and (e) 5-year OS and predicting (b) 1-year, (d) 3-year, and (f) 5-year DFS in HCC patients.

Next, we used time-dependent ROC cure analysis to analyze the predictive value of risk signature for HCC patients. As were shown at Figure 5, the AUC of risk signature for predicting 1-, 3-, and 5-year OS was 0.765, 0.73, and 0.678, respectively, which exhibited better predictive efficiency compared to TNM stage, *YTHDF2*, *YTHDF1*, *METTL3*, *KIAA1429*, and *ZC3H13* (Figures 5(a), 5(c), and 5(e)). Likewise, the AUC of risk signature for predicting 1-, 3-, and 5-year DFS was 0.695, 0.643, and 0.68, respectively, which also showed better predictive accuracy than TNM stage, *YTHDF2*, *YTHDF1*, *METTL3*, *KIAA1429*, and *ZC3H13* (Figures 5(b), 5(d), and 5(f)).

### 3.5. Validation of Risk Signature

**Figure 6:**
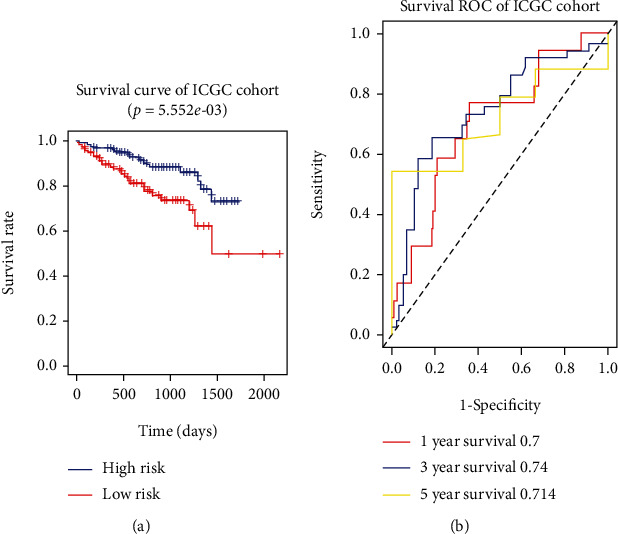
External validation of the applicability of the signature in ICGC HCC cohort. (a) Kaplan-Meier analysis of OS of patients of high-risk subgroup and low-risk subgroup in ICGC cohort. (b) AUC of risk signature in predicting 1-year, 3-year, and 5-year OS in HCC patients.

To independently test the applicability of the signature, 232 HCC patients with available OS information from the ICGC portal (https://dcc.icgc.org/projects/LIRI-JP) were further used to examine the applicability of the signature. Risk score for every patient was computed. Similarly, the signature could effectively stratify high-risk HCC patients with poorer OS and low-risk patients with better OS (HR = 2.309, 95% CI: 1.302-4.369, and *P* = 0.006; Figure 6(a)). Moreover, the AUC of risk signature for predicting 1-, 3-, and 5-year OS was 0.7, 0.74, and 0.714 (Figure 6(b)), respectively, which convincingly suggested the good discrimination and prediction of our signature.

### 3.6. Correlation of Risk Signature with Tumor-Infiltrating Immune Cells in TCGA and ICGC HCC Cohort

**Figure 7:**
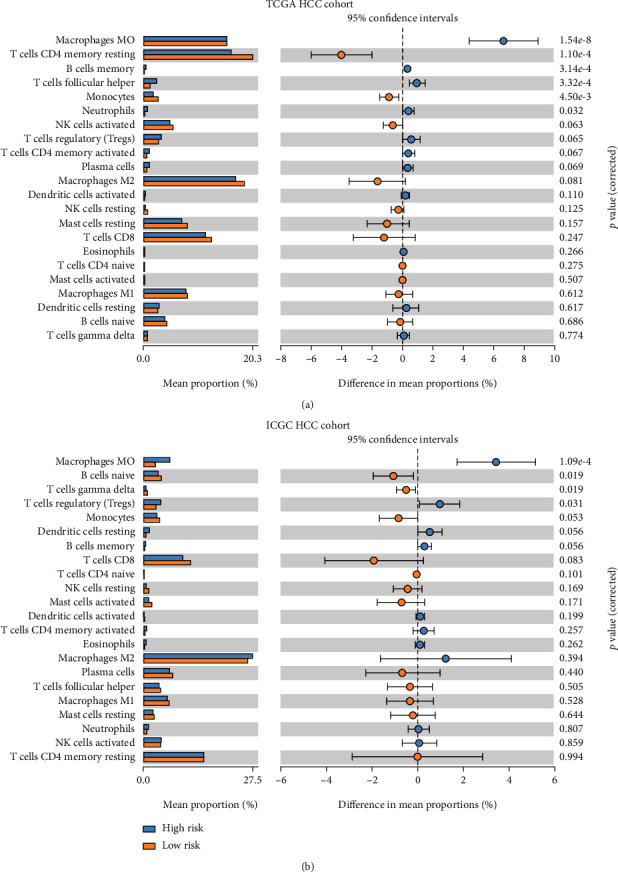
Correlation of risk signature with tumor-infiltrating immune cells in TCGA and ICGC HCC cohort. Difference of 22 kinds of infiltrating immune cells between patients with different risk scores of (a) TCGA HCC cohort. Difference of 22 kinds of infiltrating immune cells between patients with different risk scores of (b) ICGC HCC cohort.

CIBERSOR was used to calculate 22 kinds of infiltrating immune cells in patients with different risk scores. In TCGA HCC cohort, significantly higher proportions of macrophages M0 cells, memory B cells, follicular helper T cells, and neutrophils were found to be enriched in HCC patients with high risk score, while significantly higher proportions of resting memory CD4 T cells and monocytes were found to be enriched in HCC patients with low risk score (all *P* < 0.05, Figure 7(a)). In ICGC HCC cohort, significantly higher proportions of macrophages M0 cells and Treg cells were found to be enriched in HCC patients with high risk score, while significantly higher proportions of naive B cells and gamma delta T cells were found to be enriched in HCC patients with low risk score (all *P* < 0.05, Figure 7(b)). These results suggested that the risk signature was significantly associated with tumor-infiltrating immune cells, and different kinds of infiltrating immune cells in patients with different risk scores might contribute to their different prognosis.

### 3.7. Risk Signature as Indicator in Sorafenib Treatment Response for HCC Patients

**Figure 8:**
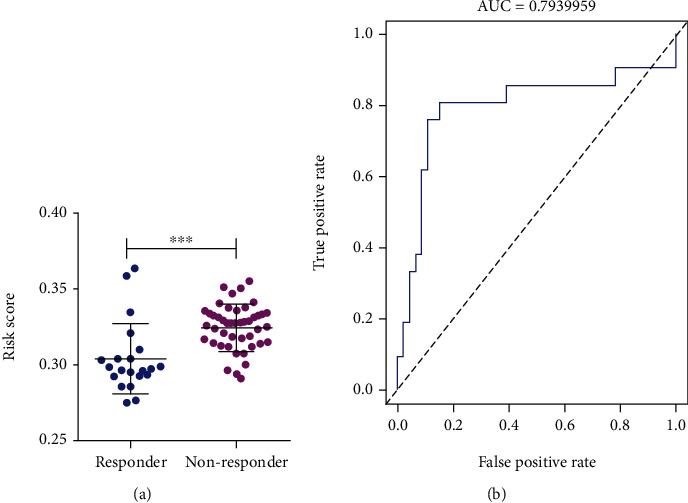
Association of risk signature with sorafinib treatment response of GSE109211 cohort. (a) Difference of risk score between sorafinib treatment responders and nonresponders. (b) AUC of risk signature in predicting in sorafinib treatment response.

To investigate the association between risk signature and sorafenib treatment response, we calculated risk score for each HCC patients treated with sorafenib of GSE109211, which contained 21 sorafenib treatment responders and 46 nonresponders. Significantly lower risk scores were found at sorafenib treatment responders compared to those in nonresponders (*P* < 0.001, Figure 8(a)). Moreover, the AUC for predicting sorafenib treatment response was 0.794 (Figure 8(b)). Taken together, the risk signature might be served as an indicator for sorafenib treatment response in HCC patients.

### 3.8. Correlation of Risk Signature with Anti-PD-1 Immunotherapy

**Figure 9:**
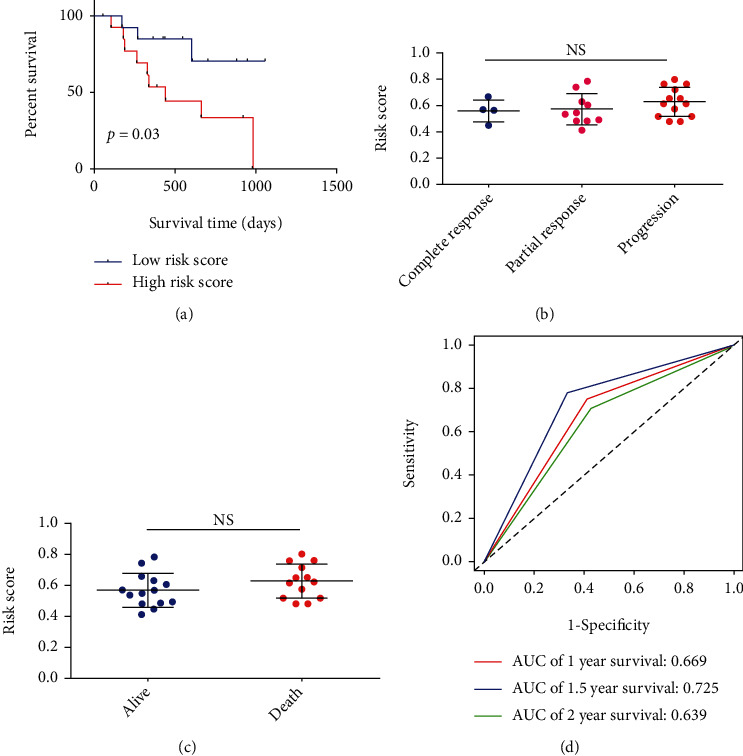
Association of risk signature with anti-PD-1 immunotherapy treatment response of GSE78220 cohort. (a) Kaplan-Meier analysis of OS of anti-PD-1 immunotherapy-treated patients with different risk scores. (b) Difference of risk score among complete anti-PD-1 immunotherapy response, partial anti-PD-1 immunotherapy response, and no anti-PD-1 immunotherapy response. (c) Difference of risk score between alive patient with anti-PD-1 immunotherapy and dead patients with anti-PD-1 immunotherapy. (d) AUC of risk signature in predicting1-year, 1.5-year, and 2-year OS in patients with anti-PD-1 immunotherapy response.

As a major breakthrough in cancer therapy, immunotherapies represented by immunological checkpoint blockade (PD-1/L1 and CTLA-4) proved promising clinical efficacy, and previous study proved that combination treatment with anti-PD-1 antibodies and sorafenib exhibited a more potent antitumor effect, but only a small number of patients could achieve durable responses [31, 32], so in the present study, we also explored whether the risk signature could predict patients' response to immune checkpoint blockade therapy in an anti-PD-1 cohort of GSE78220. Encouragingly, patients with lower risk score had better OS than patients with higher risk score (HR = 3.81, 95% CI: 1.13-11.08, and *P* = 0.03; Figure 9(a)). Besides, despite there was no statistical difference, lower risk score was found at patients with complete immunotherapeutic response compared to that in patients with partial response and patients with no response, and lower risk score was also found in alive patients treated with anti-PD-1 than that in patients of death, which might due to the limitation number of patients in the cohort (Figures 9(b) and 9(c)). Moreover, the AUC of the risk signature for predicting 1 year-, 1.5-year, and 2-year OS of patients with anti-PD-1 immunotherapies was 0.669, 0.725, and 0.639 (Figure 9(d)). In a word, the above results strongly indicated that risk signature was significantly correlated with response to anti-PD-1 immunotherapy, which might be used as a new biomarker for predicting the response to anti-PD-1/L1 immunotherapy.

## 4. Discussion

m6A modifications are mainly controlled by methyltransferases and binding proteins and [13]. Studies have reported the conservative role and mechanism of m6A modification-related genes in regulating RNA modification, but only a few literatures have studied the role of m6A modification-related genes in HCC patients. Zhao et al. found that *YTHDF1* was significantly upregulated in HCC and positively correlated with pathology stage [24]. Cheng et al. also reported that the expression of *KIAA1429* was higher in HCC and HCC cell lines, and *KIAA1429* could regulate the progression of HCC by regulating ID2 m6A modification [26]. Chen et al. discovered that *METTL3* was significantly upregulated in HCC. Knockdown of *METTL3* was also found to suppress the tumorigenicity and progression of HCC through *YTHDF2*-dependent posttranscriptional silencing of SOCS2 [25]. Moreover, Yang et al. found that *YTHDF2* was significantly related to malignancy of HCC, and miR-145 could inhibit the tumorigenicity of HCC by decreasing *YTHDF2* [33]. Collectively, these results indicated that m6A modification-related genes promoted the tumorigenesis of HCC.

Whether expressions of m6A modification-related genes could be considered as prognostic biomarker is one of the trending research topics in m6A modification research [20]. Upregulation of *YTHDF1* and *METTL3* expression was found to be related to poorer OS of HCC patients [24, 25, 27]. Similarly, in our study, *THDF1*, *HNRNPC*, *RBM15*, *METTL3*, and *YTHDF2* were independent prognostic factors for OS and DFS in HCC patients. Next, a risk signature based on the expression of five genes could differentiate HCC patients into high-risk patients with poorer OS and DFS and low-risk patients with better OS and DFS. Interestingly, this risk signature together showed better predictive efficiency in predicting OS and DFS than TNM stage or any single gene estimation alone. Therefore, this risk signature might be an advantageous method for individualized therapeutic strategies in HCC patients. In addition, we also found that the risk signature was significantly associated with tumor-infiltrating immune cells, which might influence prognosis of patients with different risk scores. Significantly higher proportions of macrophages M0 cells, neutrophils, and Treg cells were found to be enriched in HCC patients with high risk scores. Previous studies showed that macrophages could be recruited to tumor tissues and become proangiogenic cells, which were significantly associated with microvessel density and poor OS and DFS of HCC [34, 35]; Zhou et al. also found that tumor-associated neutrophils could promote the progression of HCC and resistance to sorafenib by recruiting macrophages and Treg cells [36]. These results might partly explain the reason for poorer OS and DFS in HCC patients with high risk score. Moreover, significantly higher proportions of memory CD4 T cells, gamma delta T cells, and naive B cells were found to be enriched in HCC patients with low risk score, suggesting higher proportions of infiltrated T cells and B cells. Garnelo et al. found that the degree of infiltrated T cells and B cells of tumor tissues significantly related with the improved prognosis of HCC patients [37], which might also partly explain the reason for longer OS and DFS in HCC patients with low risk score.

As an oral multikinase inhibitor, sorafenib is one of the standard care therapies for advanced stage HCC patients approved by FDA. It can prolong the survival time of HCC patients by inhibiting cell proliferation and angiogenesis and promoting cell apoptosis through inhibiting a variety of intracellular and cell surface kinases (such as c-raf, BRAF, and RET), vascular endothelial growth factor receptor (VEGFR), and platelet-derived growth factor receptor (PDGFR) [38, 39]. However, some studies have also found that HCC rapidly became sorafenib-resistant, and only about 30% of the patients could benefit from sorafenib treatment, which might greatly limit the wide clinical application of sorafenib [40, 41]. Besides, as a major breakthrough in cancer therapy, immunotherapies represented by immunological checkpoint blockade (PD-1/L1 and CTLA-4) proved promising clinical efficacy, and previous study proved that the combination treatment with anti-PD-1 antibodies and sorafenib exhibited a more potent antitumor effect, but only a small number of patients could achieve durable responses [31, 32], so identifying the HCC patients suitable for sorafenib treatment or anti-PD-1 immunotherapy or their combination therapy might be urgent and clinically significant. Encouragingly, in the present study, we found the m6A-related risk signature was significantly correlated with response to sorafenib treatment and anti-PD-1 immunotherapy. Significantly lower risk scores were found at sorafenib treatment responders or anti-PD-1 immunotherapy responders, and anti-PD-1 immunotherapy-treated patients with lower risk score had better OS than patients with higher risk score, which strongly indicated that the risk signature might be used as a new biomarker for predicting the response to sorafenib treatment and anti-PD-1 immunotherapy and even the combination of them. But independent prospective studies with a larger sample size were still needed to confirm our findings.

Though the risk signature exhibited good performance for the prognosis of HCC, several limitations should be addressed. First of all, although the prognostic value of the risk signature has been validated in external cohort, independent cohorts consist of more HCC patients were required to further verify the model. Secondly, we did not explore the potential biological functions and pathways of risk signature. The experiment in vitro and in vivo should be carried out to uncover the relevant mechanisms. Finally, previously, Huang et al. suggested that the significant expression of m6A modification-related genes was found in circulating tumor cells (CTCs) [42]. Further studies were needed to examine whether these m6A modification-related genes could be detected in peripheral blood in HCC patients and whether the risk signature in blood could still have good prognostic value.

In conclusion, *THDF1*, *HNRNPC*, *RBM15*, *METTL3*, and *YTHDF2* were independent prognostic factors for OS and DFS in HCC patients. A risk signature developed with the expression of *YTHDF2*, *YTHDF1*, *METTL3*, *KIAA1429*, and *ZC3H1* could improve the prediction of prognosis and correlate with sorafenib treatment and anti-PD-1 immunotherapy response.

## Figures and Tables

**Table 1:** Basic characteristics of 365 HCC patients from TCGA.

Variables	HCC patients (*N* = 365)
Gender (male/female)	246 (67%)/119 (33%)
Age (years, ≤60/>60)	173 (47%)/192 (53%)
Histologic grade (G1+G2/G3+G4/NA)	230 (63%)/130 (36%)/5 (1%)
T stage (T1+T2/T3+T4/NA)	271 (74%)/91 (25%)/3 (1%)
N stage (N0/N1/NA)	248 (68%)/4 (1%)/113 (31%)
M stage (M0/N1/NA)	263 (72%)/3 (1%)/99 (27%)
TNM stage (stage1+II/stage III+IV/NA)	254 (70%)/87 (24%)/24 (6%)

## Data Availability

The data of the study are available from the corresponding web page link, including GDC Data portal (https://cancergenome.nih.gov/), ICGC portal (https://dcc.icgc.org/projects/LIRI-JP), and GEO database (https://www.ncbi.nlm.nih.gov/geo/).

## Abbreviations

HCC: Hepatocellular carcinoma
m6A: N6-methyladenosine
OS: Overall survival
DFS: Disease-free survival
LASSO: Least absolute shrinkage and selection operator
GSC: Glioblastoma stem cell
VEGFR: Vascular endothelial growth factor receptor
PDGFR: Platelet-derived growth factor receptor
CTCs: Circulating tumor cells.

## Supplementary Materials

Supplementary Materials Supplementary figure 1: association of m6A modification-related genes with OS of HCC patients. Univariate analysis of mRNA expression of m6A modification-related genes with OS of HCC patients (A). Multivariate analysis of mRNA expression of m6A modification-related genes with OS of HCC patients (B-J). Gender: male vs. female; age: >60 vs. ≤60; grade: G3+G4 vs. G1+G2; T: T1 vs. T0; N: N1 vs. N0; M: M1 vs. M0; TNM stage: stage III+IV vs. stage I+II. Supplementary figure 2: relationship of m6A modification-related genes with DFS of HCC patients. Univariate analysis of mRNA expression of m6A modification-related genes with DFS of HCC patients (A). Multivariate analysis of mRNA expression of m6A modification-related genes with DFS of HCC patients (B-H). Gender: male vs. female; age: >60 vs. ≤60; grade: G3+G4 vs. G1+G2; T: T1 vs. T0; N: N1 vs. N0; M: M1 vs. M0; TNM stage: stage III+IV vs. stage I+II.
