# Supplementary figures and images for: Identification of an m6A-Related Signature as Biomarker for Hepatocellular Carcinoma Prognosis and Correlates with Sorafenib and Anti-PD-1 Immunotherapy Treatment Response
Source: Dis Markers. 2021 Jun 10;2021:5576683. doi: 10.1155/2021/5576683 (PMC8213471; doi:10.1155/2021/5576683)

**Supplementary figure 1**

**
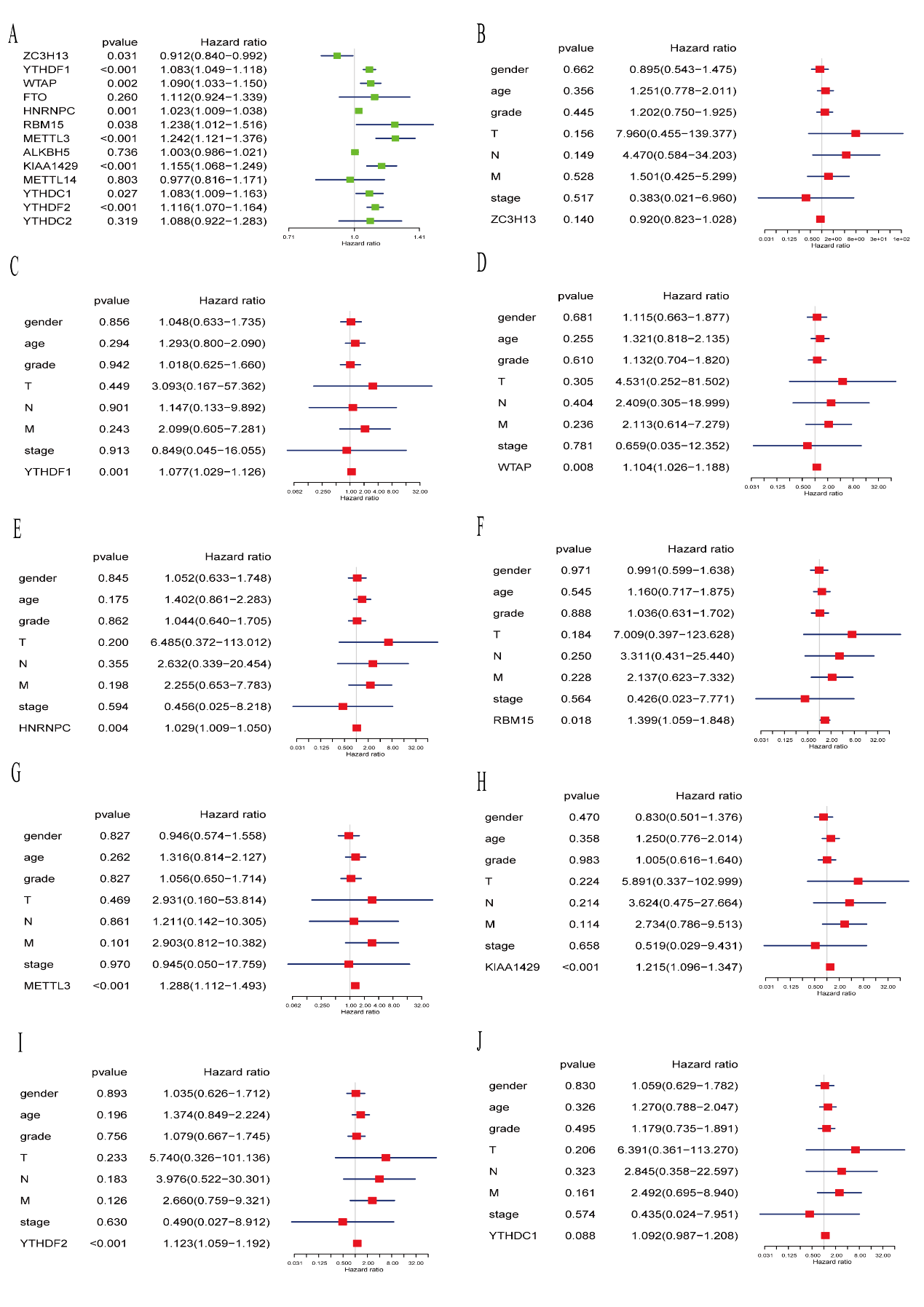
**

**Supplementary figure 2**

**
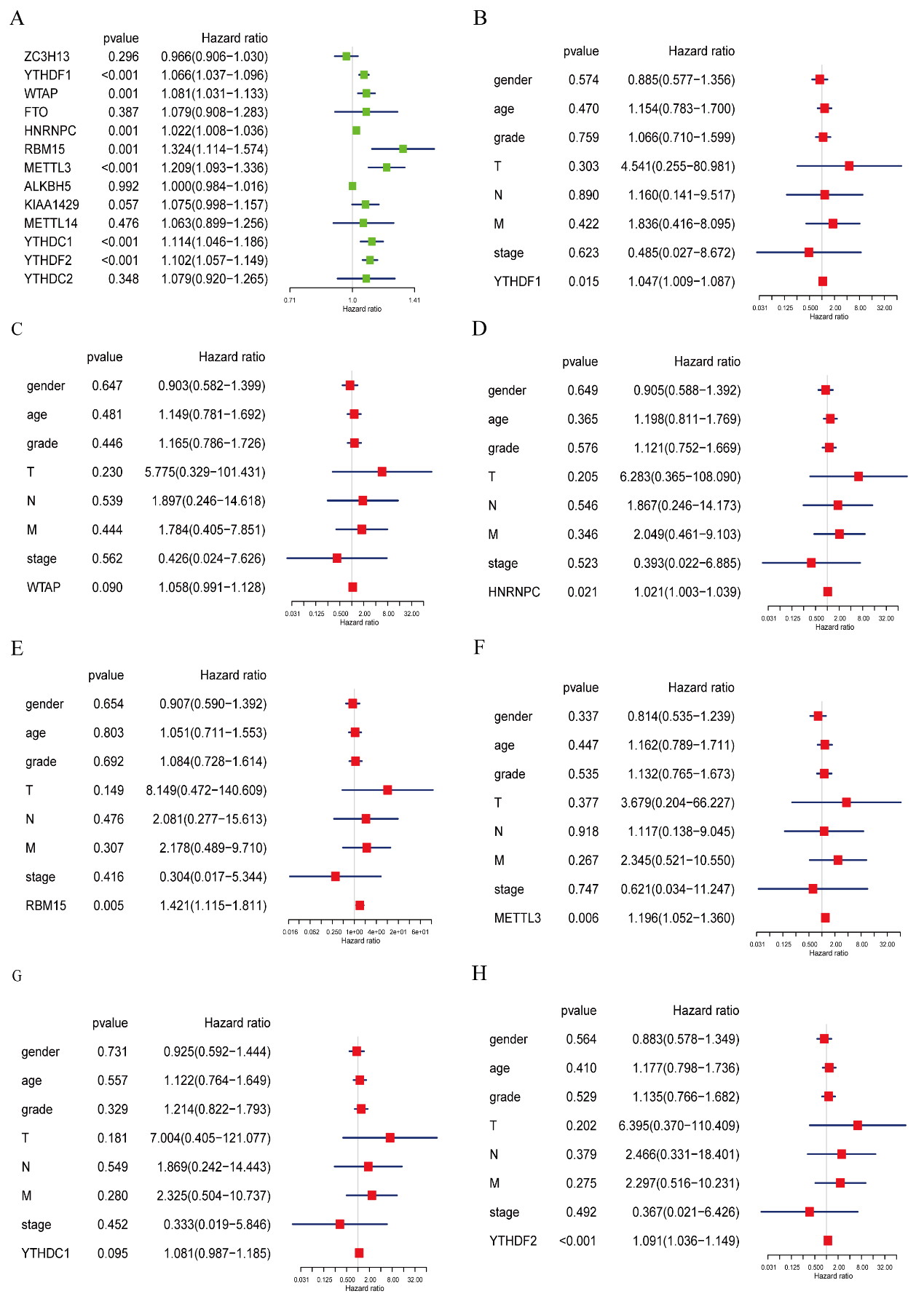
**

Supplement: Supplementary Materials — Supplementary figure 1: association of m6A modification-related genes with OS of HCC patients. Univariate analysis of mRNA expression of m6A modification-related genes with OS of HCC patients (A). Multivariate analysis of mRNA expression of m6A modification-related genes with OS of HCC patients (B-J). Gender: male vs. female; age: >60 vs. ≤60; grade: G3+G4 vs. G1+G2; T: T1 vs. T0; N: N1 vs. N0; M: M1 vs. M0; TNM stage: stage III+IV vs. stage I+II. Supplementary figure 2: relationship of m6A modification-related genes with DFS of HCC patients. Univariate analysis of mRNA expression of m6A modification-related genes with DFS of HCC patients (A). Multivariate analysis of mRNA expression of m6A modification-related genes with DFS of HCC patients (B-H). Gender: male vs. female; age: >60 vs. ≤60; grade: G3+G4 vs. G1+G2; T: T1 vs. T0; N: N1 vs. N0; M: M1 vs. M0; TNM stage: stage III+IV vs. stage I+II. [file 5576683.f1.docx]
